# Comparison of Gene Expression Profiles of Uropathogenic *Escherichia Coli* CFT073 after Prolonged Exposure to Subinhibitory Concentrations of Different Biocides

**DOI:** 10.3390/antibiotics8040167

**Published:** 2019-09-27

**Authors:** Małgorzata Ligowska-Marzęta, Viktoria Hancock, Hanne Ingmer, Frank M. Aarestrup

**Affiliations:** 1 Department of Bacteria, Parasites and Fungi, Statens Serum Institut, 2300 Copenhagen, Denmark; malm@ssi.dk; 2 Research Group for Genomic Epidemiology, National Food Institute, Technical University of Denmark, 2800 Kgs. Lyngby, Denmark; 3 Renal Research & Innovation, Baxter International Inc., SE-220 10 Lund, Sweden; viktoria_hancock@baxter.com; 4 Department of Veterinary and Animal Sciences, University of Copenhagen, 1870 Frederiksberg, Denmark; hi@sund.ku.dk

**Keywords:** biocides, uropathogenic *Escherichia coli*, transcriptional response, polymyxin

## Abstract

Biocides are chemical compounds widely used for sterilization and disinfection. The aim of this study was to examine whether exposure to subinhibitory biocide concentrations influenced transcriptional expression of genes that could improve a pathogen’s drug resistance or fitness. We used DNA microarrays to investigate the transcriptome of the uropathogenic *Escherichia coli* strain CFT073 in response to prolonged exposure to subinhibitory concentrations of four biocides: benzalkonium chloride, chlorhexidine, hydrogen peroxide and triclosan. Transcription of a gene involved in polymyxin resistance, *arnT*, was increased after treatment with benzalkonium chloride. However, pretreatment of the bacteria with this biocide did not result in cross-resistance to polymyxin in vitro. Genes encoding products related to transport formed the functional group that was most affected by biocides, as 110 out of 884 genes in this category displayed altered transcription. Transcripts of genes involved in cysteine uptake, sulfate assimilation, dipeptide transport, as well as cryptic phage genes were also more abundant in response to several biocides. Additionally, we identified groups of genes with transcription changes unique to single biocides that might include potential targets for the biocides. The biocides did not increase the resistance potential of the pathogen to other antimicrobials.

## 1. Introduction

Biocides are chemical compounds widely used for a range of purposes, such as surface and water disinfection, sterilization of medical devices, skin antisepsis, and preservation of different formulations [1]. The chemical groups quaternary ammonium compounds (QACs), biguanides, phenols and peroxides are among the most commonly used in health care [2]. Their different modes of action have been characterized and summarized previously [3,4]. Briefly, each group of biocides acts on targets located in different parts of a cell, causing diverse effects. QACs cause generalized membrane damage of the phospholipid bilayers, phenols are known to cause membrane leakage, whereas hydrogen peroxide causes DNA strand damage [3]. The action of chlorhexidine, a member of the biguanide group, is concentration-dependent and while at low concentrations it affects membrane integrity, at high concentrations it leads to congealing of cytoplasm. Even though the general mechanisms of action of these compounds on bacterial cells are known, knowledge is still lacking about the specific modes of action and bacterial targets of biocides, in particular at subinhibitory concentrations [3,4]. Among the commonly used biocides, triclosan is the only one known to have a specific bacterial target, i.e., the *fabI* gene encoding enoyl-acyl carrier protein reductase involved in fatty acid synthesis [5]. By binding to the FabI protein, triclosan inhibits fatty acid formation [6]. While most of the biocides, used at recommended concentrations, act on multiple targets in a bacterial cell [4], their action might be more selective at subinhibitory concentrations and even lead to gradual development of biocide resistance [3,7,8,9,10].

In a hospital setting, exposure of bacteria to residual concentrations of biocides could occur for example when residues of a compound are left on a surface after disinfection. Such exposure could potentially lead to development of increased resistance towards the biocide in use, as well as cross-resistance towards other antimicrobials, including antibiotics [11,12,13,14,15]. The exposure of bacteria to subinhibitory biocide concentrations could also induce other responses. In *Listeria monocytogenes*, exposure to sublethal concentration of QACs has led to increase in expression of virulence genes *prfA* and *inlA* [16]. The PrfA regulator protein in *L. monocytogenes* activates an array of virulence genes necessary for host cell infection as a result of detected changes in the environment [17]. The internalin InlA is one of the major virulence factors that, together with InlB, trigger phagocytosis and thereby enable the intracellular cell cycle of *L. monocytogenes* [18]. Several examples of increased expression of efflux pump related genes were also observed in other pathogens, such as specific *mexCD-oprJ* induction in *Pseudomonas aeruginosa* in response to subinhibitory levels of benzalkonium chloride and chlorhexidine digluconate, but not in response to other cytotoxic agents [19]. In *Staphylococcus aureus* single and multiple exposures to biocides such as benzalkonium chloride have led to increase in expression of multidrug efflux pumps [20]. Similarly, exposure of an opportunistic pathogen, *Stenotrophomonas maltophilia*, to triclosan, selected for mutants overexpressing the multidrug efflux pump, SmeDEF, which has simultaneously led to a decreased susceptibility to several antibiotics [21]. Long-term exposure of eight UPEC isolates to triclosan, polyhexamethylene biguanide, benzalkonium chloride and silver nitrate has led to decrease in biocide susceptibility to all the biocides tested, with triclosan causing the largest reduction in susceptibility [22]. Cross-resistance to antibiotics was also demonstrated in that study, with triclosan inducing cross-resistance to nitrofurantoin and ciprofloxacin and benzalkonium chloride to ciprofloxacin alone. Furthermore, long-term exposure of eight UPEC strains to triclosan has led to reduction in pathogenicity in 5 out of 8 isolates tested using the *G. mellonella* waxworm model. Exposure to benzalkonium chloride led to both decreased pathogenicity in 6 out of 8 isolates, as well as increased pathogenicity in one isolate, which prior to biocide treatment, was the least pathogenic.

Many studies have shown how the transcriptome of various pathogens becomes altered after treatment with low concentrations of biocides [12,23,24,25,26,27,28]. However, most of these studies investigated short-term response to sublethal or subinhibitory concentrations of biocides. In this study, we aimed to discover which transcripts were present or absent during growth in the presence of biocides in order to mimic the potential exposure of bacteria to residual disinfectant concentrations in a hospital setting. For this reason we cultivated the uropathogenic *Escherichia coli* (UPEC) for a prolonged period of time at subinhibitory concentrations of biocides before sampling for transcriptome analysis.

Urinary tract infections (UTIs) are among the most common bacterial infections, with an estimated 150 million cases occurring annually worldwide [29,30]. UPEC is responsible for 80% of UTI cases in all populations [31]. In hospital settings and nursing homes, UPEC accounts for over 1 million cases of catheter–associated UTI, the most common nosocomial infection [30]. In this study, we exposed *E. coli* CFT073 to four biocides—each representing a different chemical group with distinct properties: benzalkonium chloride (BAC), a Quaternary Ammonium Compound (QAC); chlorhexidine digluconate (CHX), a biguanide; hydrogen peroxide (H_2_O_2_), a peroxide; and triclosan (TSN), a phenol compound. We hypothesized that exposing the pathogen to biocides with different chemical properties and at subinhibitory concentrations could impact different targets in the cell and lead to changes in gene expression that could affect antibiotic resistance, fitness of the pathogen or virulence. In search for any changes that could affect virulence, we focused on true virulence genes, which products are directly involved in interactions with the host and responsible for the pathological damage, such as toxins or hemolysin [32]. 

## 2. Results

### 2.1. Subinhibitory Concentrations of Biocides

We determined the minimum inhibitory concentrations (MICs) for *E. coli* CFT073 for each biocide and defined the subinhibitory concentrations as MIC/4 (Table 1). At this concentration, most of the biocides investigated in this study did not inhibit the growth of the UPEC strain (Figure S1). It is worth noting that incubation with hydrogen peroxide at MIC/4 resulted in a lag phase (3.5 h), however, the growth rate was not affected. Incubation of *E. coli* CFT073 with triclosan at the concentration of MIC/4 resulted in a significant growth inhibition. After testing growth of the strain with a range of lower triclosan concentrations, we decided to use MIC/8 as the subinhibitory concentration for triclosan, as this concentration did not cause any growth inhibition.

### 2.2. Global Gene Expression after Exposure to Biocides

Bacteria present in hospitals on various surfaces will have unfavourable growth conditions with very limited nutrient access. In order to mimic these conditions, we used the MOPS minimal medium [33] in all experiments. Using microarrays, we compared the gene expression profiles of the cultures treated with the four biocides with those from an untreated culture.

Overall, a number of genes with significantly changed transcription (FDR < 0.10) after exposure of *E. coli* CFT073 to subinhibitory concentrations of biocides were identified after microarray analysis (Table 2). Treatment with benzalkonium chloride caused changes in transcription of the largest number of genes, i.e., 407, whereas treatment with triclosan led to changes in gene transcription of the lowest number of genes, 117. However, it should be noted that the FDR for comparison of triclosan with the control was slightly higher (0.128) than the recommended value due to technical problems with scanning of one of the three replicate chips and therefore some of the genes had to be omitted in the analysis of this sample.

In general, the number of genes with elevated transcription was higher than the number of genes with reduced transcription, however, the proportions of these for each biocide varied (Table 2). Treatment with chlorhexidine and hydrogen peroxide resulted in the largest proportion of highly transcribed genes (87.1% and 73.4%, respectively), whereas the ratios of genes with changed transcription were more evenly distributed for benzalkonium chloride and triclosan (58.5% and 53.8% of highly transcribed genes).

The transcription abundance of selected genes (*arnT, kgtP, papA*, and *papH*) was confirmed by qRT-PCR (quantitative real time reverse transcriptase PCR) for samples treated with benzalkonium chloride and triclosan (Table 3). Our qRT-PCR results confirmed both the direction of the transcriptional expression change, as well as the expression values for the samples and genes investigated.

### 2.3. Functional Analysis of Genes Affected after Biocide Exposure

**Figure 1:**
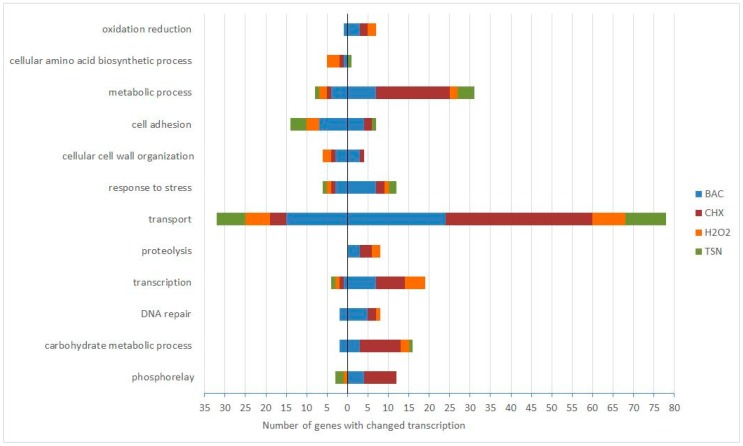
Number of genes with changed transcription following biocide treatment, grouped according to the gene ontology (GO) term biological process. The number of genes with reduced transcript levels: left side of the *y*-axis, with elevated transcript levels: right side of the *y*-axis. BAC—benzalkonium chloride, CHX—chlorhexidine, H_2_O_2_—hydrogen peroxide, TSN—triclosan. Only GO groups where transcription of more than 20 genes was elevated or reduced for all the four biocides are shown.

Twelve selected functional groups of genes, assigned according to the gene ontology group (GO) term biological process, are presented in Figure 1. Processes such as transport, transcription, and metabolism showed the largest number of genes affected by the subinhibitory concentrations of biocides used in this study. Selected groups from the category of biological process are discussed in more detail in the following paragraphs.

#### 2.3.1. Polymyxin Resistance Induced by Benzalkonium Chloride on the Transcriptional Level

Transcripts of genes involved in lipopolysaccharide modification, addition of l-Ara4N to lipid A, leading to polymyxin resistance in *E. coli* and *S.* Typhimurium [34,35,36], *arnA, arnD*, and *arnT*, were more abundant after exposure to BAC and TSN (Table 4).

Products of these genes include a decarboxylase, ArnA, a deformylase, ArnD, and a transferase, ArnT. The two latter genes are involved in the two final steps of the biosynthesis process, where the modified sugar, l-Ara4N, is added to lipid A in the outer membrane. We observed more than 2-fold higher expression for both of these genes in response to BAC (*arnD* additionally for TSN). Based on the microarray results we hypothesized that increased transcript level of the transferase, *arnT,* could result in increase of polymyxin resistance in our *E. coli* CFT073 strain. We examined the effect of a range of concentrations of Polymyxin B and benzalkonium chloride on the growth of *E. coli* CFT073 strain, as well as on a polymyxin resistant *E. coli* isolate. The MIC values for single compounds tested in this study for *E. coli* CFT073 are: MIC_PolB_ = 0.275 mg/L and MIC_BAC_ = 8 mg/L. The MIC values for the polymyxin resistant *E. coli* 2009-70-65-10 are: MIC_PolB_ = 2.8 mg/L and MIC_BAC_ = 8 mg/L.

**Figure 2:**
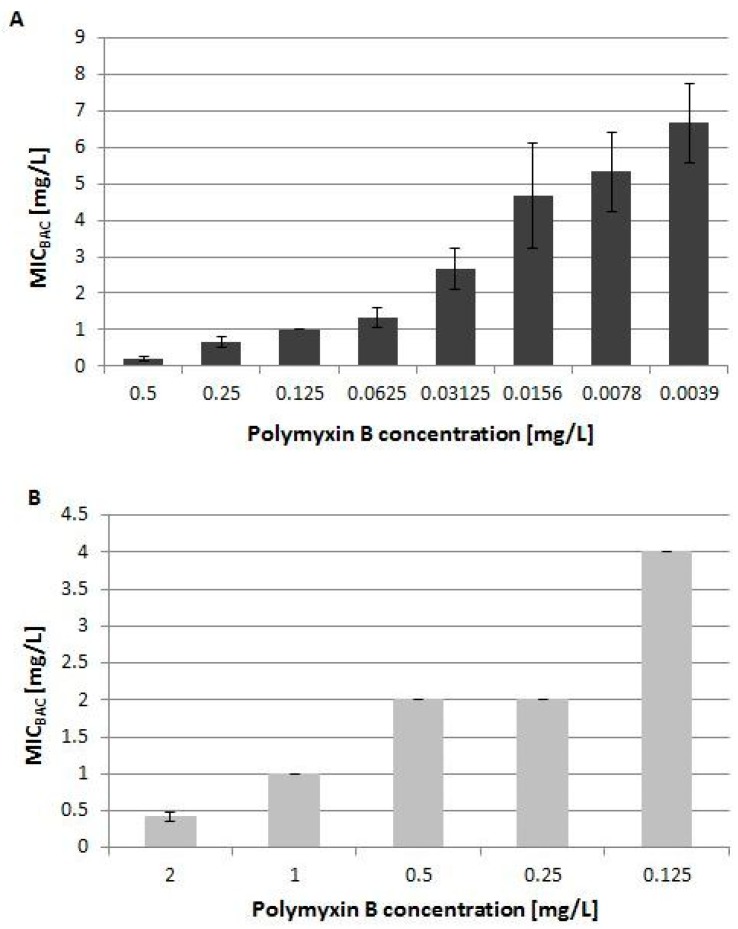
Minimum inhibitory concentrations of BAC (benzalkonium chloride) depending on the Polymyxin B concentration in the medium. (**A**) MIC values of BAC for *E. coli* CFT073, (**B**) MIC values of BAC for *E. coli* 2009-70-65-10. Data presented here are from three biological replicates. Bars represent standard errors.

We found that, under the conditions tested here, the presence of the biocide in the medium did not increase the resistance of the two examined strains to Polymyxin B using the microtiter dilution method (Figure 2) or the E-strip test. However, the results revealed that with decreasing concentration of Polymyxin B, the MIC value for BAC increased for both strains tested (Figure 2), which suggested a synergistic effect between these two compounds. Even though the transcript level of the *arnT* gene, encoding transferase responsible for the final step of lipid A synthesis was increased, the *E. coli* CFT073 strain did not exhibit a phenotype of increased resistance.

#### 2.3.2. Transport Genes are the Most Affected in Response to Prolonged Biocide Treatment

The GeneChip® *E. coli* Genome 2.0 Array (Affymetrix), used in this study, contains probe sets representing 884 genes classified into the gene ontology group 0006810 (Transport). Out of those, the transcriptional expression of 110 genes was changed by the biocides (78 with higher and 32 with lower expression levels than the control sample) and thereby this group was the most affected one. Among the genes with the highest expression in this category was *ydjN*, a gene responsible for L-cysteine uptake and for the majority of L-cystine uptake on minimal media [37,38]. The transcript level of this gene was increased almost 3-fold for hydrogen peroxide and almost 5-fold for triclosan.

The *dppABCDF* operon involved in dipeptide transport was affected by biocides. Expression of one of the genes encoding the DppABCDF dipeptide transporter (*dppC*) was elevated after exposure to benzalkonium chloride and chlorhexidine. The transcript levels of two genes from the operon *sapBCDF*, involved in putrescine export in *E. coli* increased in response to benzalkonium chloride (*sapC, sapD*), hydrogen peroxide and triclosan (*sapC*) [39]. Transcript levels of a nitrate/nitrite transporter *narU* were highly elevated for chlorhexidine (5-fold). NarU is a protein highly abundant in the stationary phase and confers a selective advantage during nutrient starvation or very slow growth [40]. Increased transcription of a gene involved in nitrate uptake after treatment with chlorhexidine could suggest activation of mechanisms similar to anaerobic respiration. The transcripts of several enzymes involved in sulfate uptake and assimilatory reduction were among the most abundant transcripts for all biocides (Table 5). Transcript abundance of some genes encoding CysAUWSbp and CysAUWCysP sulfate transporters, belonging to the ATP-Binding Cassette (ABC) superfamily of transporters [41], increased 3-fold for triclosan and between 1.67 and 4.72 fold for hydrogen peroxide after treatment.

#### 2.3.3. Variable Expression of Genes Encoding Fimbriae

We observed various changes in the transcription of genes encoding fimbrial components in response to the biocides. The gene *papA* encodes the major structural subunit of the P fimbriae, PapA, and its transcript levels decreased more than 4-fold after benzalkonium chloride treatment and almost 6-fold after triclosan treatment. Similarly, transcription of gene *papH*, encoding a protein responsible for anchoring the pilus into the membrane, PapH [42], decreased more than 2-fold for benzalkonium chloride and more than 2-fold for triclosan. These transcription changes were confirmed by qPCR (Table 3).

Among the other genes involved in cell adhesion, transcript level of uncharacterized fimbriae genes *ydeR* and *ydeS* increased in response to benzalkonium chloride almost 2-fold. Transcripts of another gene, coding for a predicted fimbrial-like adhesion protein, *yehD*, were more abundant after chlorhexidine (1.75-fold) treatment. In contrast, the transcription of *ycbR* gene (coding for a predicted periplasmic pilin chaperone) was decreased for benzalkonium chloride (−2.65-fold), chlorhexidine (−2.08-fold) and hydrogen peroxide (−2.04-fold).

#### 2.3.4. Transcription of Cryptic Phage Genes Increased in Response to Hydrogen Peroxide

The genome of *Escherichia coli* CFT073 has the size of 5,231,428 bp and contains five cryptic prophage genomes [43]. When compared with another uropathogenic *E. coli* strain, 536, which has a genome smaller by 292 kb, it is visible that the additional DNA of CFT073 contains the sequences of five cryptic prophages along with genes encoded on large pathogenicity islands [44]. In our study, transcripts of many genes encoding cryptic phage genes were elevated in response to hydrogen peroxide but lowered by other biocides.

The five top phage genes with the most abundant transcripts in response to H_2_O_2_ in our study were: *kilW* (over 7-fold)—Kil protein of bacteriophage BP-933W; *exoW* (4.92-fold)—exonuclease of bacteriophage BP-933W; *betW* (4.55-fold) - Bet recombination protein of bacteriophage BP-933W; *ssbW* (5.09-fold)—single-stranded DNA binding protein and *gamW* (5.03-fold)—host-nuclease inhibitor protein Gam of bacteriophage, which all originate from the *E. coli* O157:H7 strain EDL933. The 2753-bp long DNA sequence containing these five genes from *E. coli* EDL933 is 98% identical to a sequence in *E. coli* CFT073.

### 2.4. Biocide Specific Response

**Figure 3:**
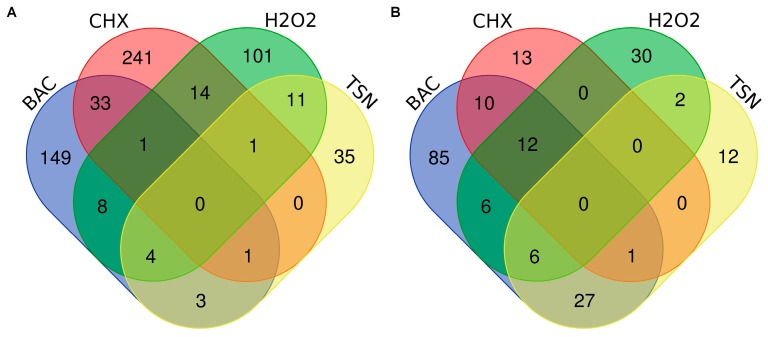
Venn diagrams showing numbers of differentially transcribed genes in response to all four biocides. (**A**) genes with increased transcripts, (**B**) genes with decreased transcripts. All genes with significantly elevated and reduced transcription were included as input, regardless of fold change. BAC—benzalkonium chloride, CHX—chlorhexidine, H2O2—hydrogen peroxide, TSN—triclosan. The graphs were drawn using a Venn diagram tool available at http://bioinformatics.psb.ugent.be/webtools/Venn/.

We used Venn diagrams to identify genes with transcripts changed in response to individual biocides, as well as to different combinations of biocides (Figure 3).

None of the genes were shared between all four biocides and in general there were few similarities between the different biocides which suggested that the response to subinhibitory concentration of each biocide affected a unique set of genes. Among the genes with elevated transcripts, the largest number was shared between benzalkonium chloride and chlorhexidine (33 genes). Among the genes with reduced transcript levels, the most genes were shared between benzalkonium chloride and triclosan (27 genes).

Table 6 presents the cellular localization of the products of the biocide specific genes. Among the genes classified as “Intracellular”, there were generally more genes with elevated than reduced transcripts for all biocides with the exception of chlorhexidine. Among the genes classified as “Membrane”, there were more genes with reduced transcripts for all of the biocides. Only for some of these genes, the two other Gene Ontology categories, “Biological process” and “Molecular function” were known, and for this reason, the “Unclassified” group of genes could include potential unknown targets of the biocides.

## 3. Discussion

In this study we have described the transcriptional response of the uropathogenic *Escherichia coli* strain, CFT073, to subinhibitory concentrations of four biocides: benzalkonium chloride, chlorhexidine, hydrogen peroxide and triclosan. In our experimental setting, we allowed the bacteria to adjust their gene expression to the subinhibitory levels of biocides by incubating them in the presence of the compounds for a total period of 22 h, including one medium transfer. This experimental setup enabled determination of which genes were transcribed by the bacterium to maintain growth in the presence of biocides, rather than to measure the immediate response to the biocides.

It needs to be noted that for our experimental setting we used the MOPS medium [33] supplemented with 0.2% glucose. Under these conditions, the expression of a number of genes is likely changed due to catabolite repression. Interestingly, the presence and type of carbon source might have an effect on the sensitivity of the bacterial cells to the biocides. In a study by Ishikawa et al., *E. coli* cells grown with glucose were less sensitive to surfactants Cetyltrimethylammonium bromide (CTAB) and N-dodecyl-*N,*N-dimethylglycine (DDMG) and had a lower respiratory activity than the cells grown with other, less favorable carbon sources, such as glycerol, succinate and acetate [45]. The authors of that study point to possible alterations of the cell envelope structure leading to reduced membrane permeability and decreased ability of the surfactants to localize at the membrane, as the reasons for the observed difference in sensitivity. It is very likely that the results of our study would be different if another carbon source had been used and it remains of interest to investigate that further.

The level of response among the four biocides varied, with benzalkonium chloride affecting transcription of the largest number of genes and with triclosan affecting the smallest number of genes. The reason for the latter could either be the fact that a lower sub-MIC concentration (0.125 × MIC) was used for triclosan, or it could be related to the more specific mechanism of action of this compound [5,6]. Our hypothesis was that the presence of biocides in the medium in which the bacteria are growing could increase transcription of genes involved in virulence, antibiotic resistance or in general increase the fitness of the pathogen.

Transcript levels of genes from the *arnBCADTEF* operon, involved in lipopolysaccharide modification leading to polymyxin resistance in *E. coli* and *S.* Typhimurium, were elevated when *E. coli* CFT073 was grown in the presence of BAC and TSN. Polymyxins belong to a group of cationic antimicrobial peptides and they owe their antimicrobial action to binding to lipid A, a component of the negatively charged lipopolysaccharide (LPS) in the outer membrane [46,47,48,49]. One of the mechanisms contributing to polymyxin resistance in Gram negative bacteria is the addition of 4-amino-4-deoxy-l-arabinose (l-Ara4N) to a phosphate group in lipid A by the ArnT transferase [49,50]. This modification decreases surface negative charge of the LPS, thus reducing polymyxin binding to the membrane [51]. Biosynthesis of l-Ara4N is a multistep process that employs all the genes from the *arnBCADTEF* operon [49,50,52]. In *Salmonella enterica* serovar Typhimurium homologous genes are encoded by the operon *pmrHFIJKLM* and were shown to contribute to the strain’s resistance against polymyxin by modifying lipid A on the LPS [53,54,55]. Overexpression of *pmrK*, encoding the homologue of *arnT*, has been shown to lead to a swarming phenotype and an increased resistance to polymyxin in *S. enterica* serovar Typhimurium [56]. Even though the transcript level of gene *arnT* was elevated in this study, we did not observe an increase in resistance to polymyxin when *E. coli* cells were incubated with this antibiotic and the biocide in vitro (Figure 2). The relative transcript change of the *arnT* gene, as well as the other two genes, *arnA* and *arnD*, was 2-fold (Table 4), which might not be enough to observe phenotypically. Another reason could be a post-transcriptional or post-translational modification of the gene product.

Functional analysis of the genes affected revealed certain possible adaptations allowing growth in the presence of biocides. Transport was the functional group where transcription of the most genes was affected. Transcripts of certain genes from the operon encoding the dipeptide transporter, DppABCDF were increased in response to benzalkonium chloride and chlorhexidine. In *E. coli* MG1655, the *dppABCDF* operon was activated after 3.5 h of growth, when the free amino acids and nucleotides got depleted from the LB medium [57]. This operon, along with others involved in the Ntr (Nitrogen regulated) response [58], therefore serve as a way of scavenging nitrogen in case of nitrogen limitation. One could speculate that, in our study, the biocides at these subinhibitory concentrations deplete the bacteria of nitrogen. The DppA protein (periplasmic dipeptide transport protein) was found to be expressed on the surface of *E. coli* CFT073 and four other reference UPEC strains during in vitro growth in human urine, identified by mass spectrometric analysis of EDTA heat-induced outer membrane vesicles (OMVs) [59].

The reason for increase in transcription of two of the genes from the operon *sapABCDF* in response to benzalkonium chloride, hydrogen peroxide and triclosan is unclear. The function of this operon in *E. coli* is to export putrescine [39]. Polyamines, such as putrescine, spermidine and spermine, are ubiquitous among microorganisms and have various roles in the cell [60]. Disruption of polyamines metabolisms results in many changes in cellular processes, such as transcription, translation, regulation of gene expression or stress resistance. In *E. coli*, deficiency in two polyamine catabolic pathways prevented growth of the strain exposed to oxidative stress and impaired its growth during heat stress and at sublethal kanamycin concentration [61]. In *S.* Typhimurium, the *sapABCDF* operon is required for virulence and resistance to antimicrobial peptides (AMPs) melittin and protamine [62,63], likely occurring by transporting the peptides from their putative targets into the cytoplasm where they get degraded. A similar mechanism was clearly demonstrated in *Haemophilus influenzae* where a strain lacking the Sap permease complex was unable to transport AMPs to the bacterial cytoplasm for degradation and accumulated them in the periplasm instead [64]. However, it was shown that the Δ*sapBCDF E. coli* strain did not affect resistance to antimicrobial peptide LL-37 [39]. In another study involving the *E. coli* CFT073 strain, the Sap operon was identified, through transposon mutagenesis, as one of the factors required for optimal fitness in a mouse model of invasive UPEC infection, as well as involved in protection against AMPs such as Polymyxin B [65]. 

Among the highest transcribed genes were the ones involved in sulfate assimilation pathway. Sulfur is an essential element, as it is the building block of many biomolecules in a bacterial cell, such as the amino acids cysteine and methionine or cellular cofactors such as biotin and iron-sulfur clusters [66]. Cysteine is a component of the compounds glutathione and thioredoxin, which are important for maintaining redox homeostasis in the cell [67,68]. Methionine plays a role as a starting point of cycles involved in polyamines biosynthesis and in translation of mRNA into proteins [69]. Similar upregulation of sulfur assimilation genes was observed in an ethanologenic *E. coli* strain, LY180, used for the fermentation of sugars in hemicellulose hydrolysates, as a result of addition of furfural, a toxic side product of sugar fermentation that inhibits microbial growth [70]. Authors of that study explain that conversion of sulfate to hydrogen sulfide is an energy costly process, requiring four molecules of NADPH. They conclude that addition of furfural most likely results in an intracellular deficit in sulfur-containing amino acids, such as cysteine and methionine and hence, upregulation of sulfate uptake genes. In that study, however, also genes involved in methionine synthesis and uptake of an alternative sulfate source, taurine, were upregulated. In our study, only transcription of cysteine genes increased in response to benzalkonium chloride, hydrogen peroxide and triclosan. In general, this significant increase in transcription of sulfate transporters, as well as genes involved in biosynthesis of molecules during growth, indicates the need for sulfur for biosynthesis of molecules in the presence of certain biocides.

Uropathogenic *E. coli* encodes a repertoire of fimbriae that are necessary for establishment of infection [71]. Among the ones helpful in the colonization of the host are P fimbriae, type 1 fimbriae, as well as F1C, S, M, and Dr fimbriae [72,73]. It has been suggested that each of these fimbriae types plays a different role during the different stages of infection and it has been shown that the expression of type 1 fimbriae is inversely coordinated with the P fimbriae expression [74]. It is also known that the fimbriae are expressed differently depending on external factors such as temperature, medium, pH, and osmolarity [75,76]. In our study, trancripts of both *papA* and *papH* genes were reduced more than 2-fold in response to benzalkonium chloride and triclosan. A similar downregulation of the *papA* gene in response to low concentration of triclosan had been reported previously when triclosan’s effectiveness against uropathogens was examined in ureteral stents in vitro [77], however, transcript decrease of *papA* after treatment with benzalkonium chloride has not been reported before. Such reduction, in response to these two biocides, indicates that these two compounds are capable of downregulating virulence factors, even at low concentrations.

Interestingly, in our study we observed varying transcription changes among genes encoding putative fimbriae in response to some of the biocides, in addition to transcript reduction of the *papA* and *papH* gene in response to benzalkonium chloride and triclosan. We found transcripts of a gene coding for a fimbrial-like adhesion protein, *yehD*, as well as the uncharacterized fimbrial genes *ydeR* and *ydeS* to be increased in response to two of the biocides, whereas transcripts of a gene coding for a predicted periplasmic pilin chaperone, *ycbR*, were reduced in response to three of the biocides. One study attempted to characterize the *yeh* operon together with six other operons encoding putative adhesins in *E. coli* K-12 strain [78]; it was demonstrated that while these fimbriae were poorly expressed in laboratory conditions, they were functional when expressed from a constitutive promoter and they promoted adhesion to abiotic and epithelial cell surfaces. These fimbriae were also shown to be activated by carbon catabolite repression and, additionally, regulated by the global transcription repressor, H-NS. The authors concluded that the expression of the investigated fimbriae, as a result of environmental challenges, could allow *E. coli* to better adapt to and colonize different ecological niches. Similarly, we hypothesize that this differential transcription of fimbriae genes *pap*, *yehD*, and *ycbR* observed in our study could contribute to our strain’s ability to adapt to the conditions created in the presence of biocides.

Although transcription of cryptic phage genes increased in response to hydrogen peroxide, it was lowered in response to the other biocides tested. Hydrogen peroxide is a known inducer of phage genes in different bacteria and this has been demonstrated in *E. coli* O157:H7 [79], *Streptococcus* [80], and *S. enterica* serovar Typhimurium LT2 [81]. A study investigating the role of cryptic prophages during different types of stress in *E. coli* K-12 revealed that the cryptic prophages increase resistance to sublethal concentrations of quinolone and β-lactam antibiotics, primarily by inducing proteins that inhibit cell division [82]. The prophages were also important for withstanding osmotic, oxidative and acid stresses, increasing growth and influencing biofilm formation. Wang et. al., suggest that fossil phage genes may be important for bacteria to increase their fitness and they found two proteins mainly responsible for this, KilR and DicB, both inhibiting cell division. One could speculate that the transcription increase of cryptic phage genes of the *E. coli* strain CFT073 in response to H_2_O_2_, observed in our study, might have played a role in regaining growth after a 3.5 h lag-phase.

Our study compared the response of one pathogen to subinhibitory concentrations of four different biocides under the same conditions. Biocides are known to affect a broad range of targets in the bacterial cell and so far, in addition to a common core response, a species-specific response has been described when comparing transcriptomes of *Escherichia coli* and *Salmonella* Typhimurium in response to MIC of triclosan [23]. Similarities and differences in response to biocides among different bacterial species have been extensively discussed elsewhere [83]. According to our knowledge, no study has compared the transcriptional response of the same bacterium to different biocides. This approach allowed us to identify genes that are affected in the uropathogenic *E. coli* strain CFT073 uniquely in response to single biocides. Future work could include comparing responses of different pathogens to the same biocides in order to identify a “core” response to each biocide across many species.

## 4. Materials and Methods 

### 4.1. Chemicals and Reagents

The biocides used in this study included benzalkonium chloride (BAC, 50%, Alfa Aesar), chlorhexidine digluconate (CHX, 20% (*w/v*), AlfaAesar), hydrogen peroxide (H_2_O_2_, 30%, Fluka) and triclosan (TSN (Irgasan), Sigma-Aldrich). Solutions of H_2_O_2_ at appropriate concentrations were freshly prepared before each experiment and the following stock solutions of the other biocides were used throughout the whole study: BAC (5120 mg/L), CHX (1280 mg/L), TSN (300 mg/L).

### 4.2. Bacterial Strains and Growth Conditions

The uropathogenic *E. coli* CFT073 strain was isolated from a patient with acute pyelonephritis [84]. A polymyxin resistant *E. coli* strain 2009-70-65-10 was isolated from food products during the 2009 DANMAP screening. All strains were cultivated at 37 °C on Lysogeny broth (LB) agar plates and grown in liquid culture with shaking (200 rpm) in a slightly modified MOPS (morpholinepropanesulfonic acid) minimal medium [33] (19 mM NH_4_Cl and 0.552 mM K_2_SO_4_ were used in this study), supplemented with 0.2% glucose and 0.5% casamino acids.

### 4.3. Determination of MIC Values of Biocides Using the Broth Microdilution Method

To determine the MIC (minimum inhibitory concentration) value for the compounds tested in this study, we used the broth microdilution method according to the Clinical & Laboratory Standards Institute’s (CLSI) guidelines [85]. Several colonies of a freshly cultivated strain were suspended in 0.9% NaCl solution to a concentration of 1-2 × 10^8^ CFU/mL, adjusted using McFarland reagent of density 0.5. Each well in a 96-well polystyrene plate (Nunclon Δ surface, cat. no 143761) was filled with 100 μL of MOPS medium containing approximately 5 × 10^5^ CFU/mL per well. The biocides and polymyxin were diluted in MOPS medium and added to the wells so that two-fold dilutions of the compound were obtained in each column. Each plate contained growth control wells and sterile control wells and MIC measurement of each compound was performed in triplicates. Plates were incubated at 37 °C in a static incubator and the results were read after 16–20 h. The MIC values reported here were the lowest concentrations of the compounds tested that resulted in no visible growth (Table 1).

### 4.4. Collecting RNA Samples for Microarray Analysis

A freshly restreaked colony of *E. coli* CFT073 was incubated for 16–18 h in 2 mL MOPS medium in a shaking incubator (200 rpm). Six flasks with 10 mL MOPS medium were then inoculated with that culture to an OD_600_ = 0.05. One of the flasks served as a control without any biocides and each of the four remaining flasks contained one of the biocides used in this study at the sub-MIC concentrations given in Table 1. The cultures were allowed to grow for 18–20 h at 37 °C with shaking and then were transferred to six flasks with 25 mL fresh MOPS medium in the same manner. After each of the cultures had grown to OD_600_ = 0.6, 2 mL was quickly transferred to a double volume of RNA Protect reagent (Qiagen). Each sample was then vortexed for 5 s, incubated for 5 min at room temperature and centrifuged for 10 min at 3214× *g* rcf (Eppendorf 5810R centrifuge with A-4-52 rotor). The supernatant was carefully removed and the pellets were stored at −20 °C until RNA extraction.

### 4.5. Samples Preparation for Microarray Analysis

The bacterial pellets were lysed using 0.2 mg lysozyme per sample and RNA was extracted using the RNeasy Mini Kit (Qiagen) according to the manufacturer’s instructions, including the on-column DNase digestion. All 18 RNA samples were visualized on 0.8% agarose gel to visually confirm lack of DNA and RNA degradation. The concentrations of samples were measured using Nanodrop 1000 (Thermo Scientific) and the A_260/280_ and A_260/230_ ratios values were inspected to determine the purity of the samples. The integrity of RNA was finally confirmed by Agilent 2100 Bioanalyzer System (Agilent Technologies) using Agilent RNA 6000 Nano Kit. Synthesis of cDNA from 10 μg of RNA per sample, labelling and hybridization to the microarray chips were performed according to the instructions in the GeneChip^®^ Expression Analysis Technical Manual version P/N 702232 revision 3 (Affymetrix). GeneChip^®^ *E. coli* Genome 2.0 Array (Affymetrix) was used for this study and the chips were scanned using GeneChip^®^ Scanner 3000.

### 4.6. Microarray Data Analysis

The DNA-Chip Analyzer (dChip) software package for probe-level and high-level analysis of gene expression microarrays and SNP microarrays was used to normalize the data and calculate the expression values (www.dchip.org) [86]. In order to make the arrays comparable, they were normalized at probe cell level using the invariant set normalization method [87]. Probe selection and computation of expression values were performed using model-based (PM-only) method. The computed expression levels were attached with standard errors and these were then used to compute 90% confidence intervals of fold changes in two-group comparisons. The three arrays hybridized with samples from *E. coli* CFT073 grown in MOPS medium without any biocides served as a baseline for identifying gene expression changes in the arrays hybridized with biocide-treated samples. Permutation was used to estimate the empirical false discovery rate (FDR) of differentially expressed genes. Permuting the samples randomly 200 times resulted in FDR values <10% with the exception of triclosan-treated samples for which FDR was 12.8%. The reason for the latter was a high number of array outliers due to problems with scanning of one of the three chips with triclosan-treated samples. These array outliers were treated as missing data in subsequent data analysis.

When comparing the biocide specific response, we used the Gene Ontology classification term “Cellular component” for each gene to determine the localization of the products of the biocide specific genes. We divided the up- and downregulated genes for each biocide into those that produce proteins acting in the membrane (this category was designated “Membrane” and included the outer membrane, the periplasmic space, and the inner membrane) and in the cytoplasm (“Intracellular”) (Table 6). Many genes were placed in the category “Unclassified”, as no Gene Ontology term from the “Cellular component” category had been assigned.

### 4.7. Quantitative Real Time PCR

Fresh samples for RNA extraction for quantitative real time RT-PCR were collected following the same protocol as for microarray sample collection, but here Qubit (Life Technologies) was used to determine RNA concentration. The primers (Table 7) were designed using Primer3 Plus [88] and tested to fulfil the assumption that the amplification efficiencies of target and reference genes should lie near 100% [89]. This assumption was tested by making standard curves based on 10-fold dilutions for each primer pair and calculating the amplification efficiencies from the equation E = 10^−1/slope^ and %Efficiency %E = (E−1) × 100% [90]. The %E values of primer pairs used in this study are shown in Table 7. Due to the fact that the expression of genes between four different treatments was compared here, we selected three different reference genes, whose expression values were unchanged in our microarrays in response to all the biocide treatments applied. These genes were *gapA* (Glyceraldehyde-3-phosphate dehydrogenase)*, idnT* (L-iodonate and D-gluconate transporter), and *accD* (Acetyl-CoA carboxylase subunit beta) and were amplified with each run.

Quantitative real time PCR was performed with 10 ng RNA per one-step reaction (reverse transcription and PCR occurred in a single tube) using the QuantiFast SYBR Green RT-PCR kit (Qiagen, cat. no 204154), according to the manufacturer’s instructions. All reactions were performed in the 7900HT Fast Real-Time PCR System (Applied Biosciences). For each sample treatment, a fitting reference gene was chosen to calculate relative expression, based on the assumption that the difference between the C_T_ value of the reference gene in the untreated and the treated sample was not more than 1.5 cycle. Melting curve analysis was performed after each run and the melting profiles of all genes were screened for the presence of by-products, such as primer-dimers or contamination. Each run included controls without template and without reverse transcriptase. Expression data from three biological replicates were collected and the relative expression of the target genes was calculated using the 2^−ΔΔCT^ (Livak) method [89].

### 4.8. Polymyxin B and Biocide Cross-Resistance

Polymyxin B sulfate (Sigma) was dispensed into polypropylene microtiter plates (Greiner Bio-One, cat. no 650261) together with benzalkonium chloride (BAC). Each row in a plate contained serial two-fold dilutions of a biocide solution and each column contained serial two-fold dilutions of Polymyxin B sulfate. The following ranges of compounds were tested in a single plate: Polymyxin B: 1–0.03125 mg/L and 0.125–0.0039 mg/L, BAC: 64–0.125 mg/L. Freshly restreaked colonies of strain *E. coli* CFT073 were resuspended in 0.9% NaCl and cell density adjusted to 1–2 × 10^8^ CFU/mL using McFarland reagent 0.5. The culture was added to the microtiter plate, resulting in 5 × 10^5^ CFU/mL in each well. Plates were sealed and incubated at 37 °C in a static incubator. Results were read after 18 h. The same procedure was applied to the Polymyxin resistant strain *E. coli* 2009-710-65-10, but here the Polymyxin B range on the plates was from 4 to 0.125 mg/L. All strains were tested in three biological replicates.

### 4.9. Microarray Data Accession Number

The microarray analysis and expression data are available in NCBI’s Gene Expression Omnibus (http://www.ncbi.nlm.nih.gov/geo) with the accession number GSE135556.

## 5. Conclusions

In summary, the data analysed in this study allow for better understanding of how the uropathogenic *E. coli* CFT073 adapts to growth at subinhibitory concentrations of biocides. Careful analysis of the data did not reveal any evidence of increased transcription of true virulence genes or antibiotic resistance as a result of treatment with the biocides tested. In addition, no cross-resistance to antibiotics could be confirmed on a phenotypical level. We could show, however, a synergistic action between polymyxin and benzalkonium chloride in vitro. The gene expression data also revealed increased transcription of genes involved in uptake of peptides, sulfate, activation of cryptic phage genes, as well as variable transcription of fimbriae—all potential indicators of this pathogen’s adaptation to growth with the four biocides. Finally, by comparing the groups of genes affected for each biocide, we found sets of biocide-specific genes, among which many could not be classified by function and are therefore potential candidates for targets of these compounds.

## Figures and Tables

**Table 1:** Minimum inhibitory concentration (MIC) and subinhibitory minimum inhibitory concentration values (sub-MIC) of *E. coli* strain CFT073 for each biocide. The values were determined from three biological replicates.

Group	Biocide	MIC	Sub-MIC
QAC	Benzalkonium chloride (BAC)	8 mg/L	2 mg/L
Biguanide	Chlorhexidine (CHX)	0.25 mg/L	0.0625 mg/L
Peroxide	Hydrogen peroxide (H_2_O_2_)	0.004%	0.001%
Phenol	Triclosan (TSN)	2.5 mg/L	0.3 mg/L

**Table 2:** Total number of genes with changed transcription (FDR < 0.10, for triclosan FDR < 0.13) for all comparisons. Numbers and percentages of genes with elevated and reduced transcripts in the presence of each biocide are presented. Abbreviations: BAC—benzalkonium chloride, CHX—chlorhexidine, H_2_O_2_—hydrogen peroxide, TSN—triclosan. “Up” and “Down” refer to direction of the observed relative transcription change.

Biocide	Number of Total Genes	Up		Down	
		Number	%	Number	%
BAC	407	238	58.5	169	41.5
CHX	389	339	87.1	50	12.9
H_2_O_2_	233	171	73.4	62	26.6
TSN	117	63	53.8	54	46.2

**Table 3:** Confirmation of fold change of selected genes by quantitative real-time PCR. Fold change values for qPCR are mean values of 2^−ΔΔCT^ obtained from three biological replicates, reported with the standard deviation values (SD). BAC—benzalkonium chloride, TSN—triclosan.

	BAC			TSN		
Gene	Microarray	qPCR	SD	Microarray	qPCR	SD
*arnT*	2.38	−0.82	2.14	−	-	−
*kgtP*	−	−	−	−1.96	−2.14	7.24
*papA*	−4.25	−4.57	2.96	−5.98	−17.31	18.97
*papH*	−2.35	−3.75	1.54	−2.41	−6.93	5.55

**Table 4:** Increased transcription of genes from the *arnBCADTEF* operon in response to three biocides. BAC—benzalkonium chloride, TSN—triclosan.

Gene	BAC	TSN	Gene Product
*arnA*	1.74	−	fused UDP-l-Ara4N formyltransferase/UDP-GlcA C-4′-decarboxylase
*arnD*	2.30	2.52	Undecaprenyl phosphate-alpha-l-ara4FN deformylase
*arnT*	2.38	−	4-amino-4-deoxy-l-arabinose transferase

**Table 5:** Fold changes of genes involved in pathways transporting or utilizing sulfur in response to subinhibitory concentrations of four biocides. BAC—benzalkonium chloride, CHX—chlorhexidine, H_2_O_2_—hydrogen peroxide, TSN—triclosan.

Gene	Fold Change	Pathway(s) or Processes
*cysH*	H_2_O_2_ (2.43), TSN (6.79)	Superpathway of sulfate assimilation and cysteine biosynthesis; Sulfate reduction I (assimilatory)
*cysI*	TSN (11.39)	
*cysJ* *cysN*	BAC (1.72) TSN (3.44)	
*cysD*	CHX (4.32), H_2_O_2_ (9.28), TSN (18.58)	Sulfate activation for sulfonation
*sbp(c4868)*	H_2_O_2_ (1.67)	Sulfate/thiosulfate/selenite transport
*cysA*	TSN (3.25)	
*cysP*	H_2_O_2_ (4.72), TSN (3.12)	

**Table 6:** Division of the biocide specific genes according to the location of their products in the cell, based on the Gene Ontology category “Cellular component”. The column “Intracellular” contains the genes that express proteins acting in the cytoplasm. The column “Membrane” contains the genes that express proteins acting in the outer membrane, periplasmic space and the inner membrane. BAC—benzalkonium chloride, CHX—chlorhexidine, H_2_O_2_—hydrogen peroxide, TSN—triclosan. “Up” and “Down” refer to direction of the observed relative transcription change.

Biocide	Intracellular		Membrane		Unclassified		Total Number of Biocide Specific Genes	
	Up	Down	Up	Down	Up	Down	Up	Down
**BAC**	55%	29%	35%	38%	10%	33%	86	70
**CHX**	29%	38%	24%	31%	47%	31%	181	16
**H_2_O_2_**	53%	20%	7%	35%	40%	45%	83	20
**TSN**	67%	16%	33%	67%	0	17%	9	6

**Table 7:** Primers used in quantitative real time PCR.

Primer Name	Primer Sequence 5’—3’	Product Size (bp)	Amplification Efficiency (From Standard Curve)	Reference
accD2_for	CTAACAGGCTATGCAGGCGA	168	109%	This study
accD2_rev	ACATTACTCCCACCCGCAAG			
gapA2_for	GTTGACCTGACCGTTCGTCT	172	111%	This study
gapA2_rev	CCGCTTTAGCATCGAACACG			
idnT2_for	CGGCGTTAATGGCTAACACG	139	105%	This study
idnT2_rev	TCACACGTAAACGACCCTGG			
arnT4_for	TTGCACTGGATGATGCCCAA	167	102%	This study
arnT4_rev	CGGCATTATCGTCCAGCTCA			
kgtP3_for	GTGAAACCAGAAACGCCACC	131	97%	This study
kgtP3_rev	ATATGCGGTCGCCAATGCTA			
papA_for_S	GTGCCTGCAGAAAATGCAGAT	88	103%	[74]
papA_rev_S	CCCGTTTTCCACTCGAATCA			
papH2_for	TAATCTGCCAGGCGTCTTCC	70	112%	This study
papH2_rev	AGGGCTGCTTTTCATGGTGA			

## Supplementary Materials

The following are available online at https://www.mdpi.com/2079-6382/8/4/167/s1, Figure S1: Representative growth curves of *E. coli* CFT073 with all four biocides at sub-MIC concentrations based on optical density (OD_600_) measurements.
