# Supplementary material for: Comparison of Gene Expression Profiles of Uropathogenic Escherichia Coli CFT073 after Prolonged Exposure to Subinhibitory Concentrations of Different Biocides
Source: Antibiotics (Basel). 2019 Sep 27;8(4):167. doi: 10.3390/antibiotics8040167 (PMC6963283; doi:10.3390/antibiotics8040167)
Supplement: Supplementary file 1 [file antibiotics-08-00167-s001.pdf]

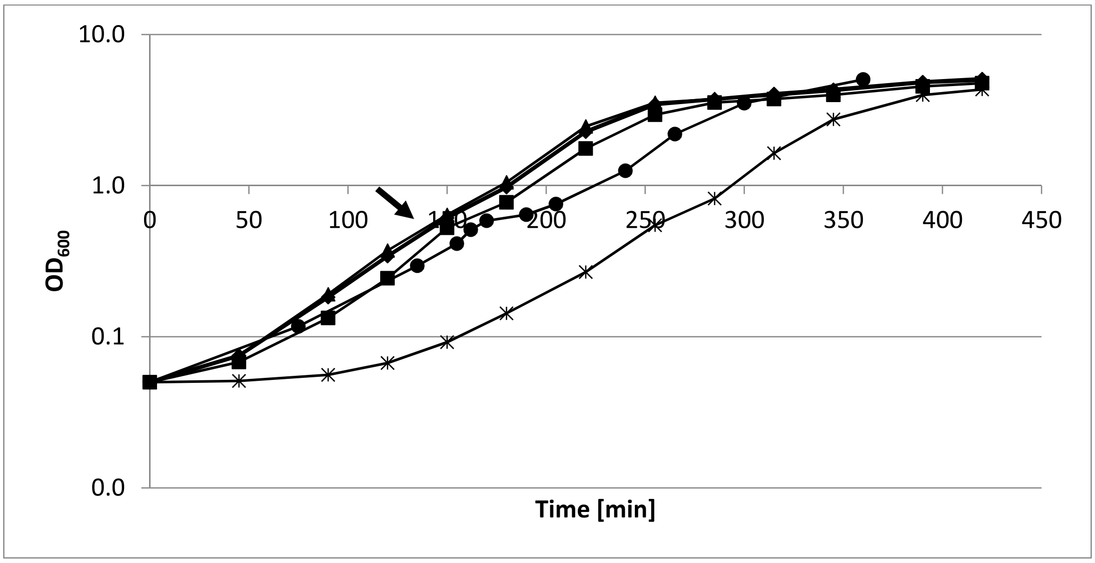

Figure S1. Representative growth curves of *E. coli* CFT073 with all four biocides at sub-MIC concentrations based on optical density (OD_{600}) measurements. *E. coli* CFT073 was grown in MOPS medium without biocides (♦) and with each of the f**our** biocides at the concentration of MIC/4: benzalkonium chloride (■), chlorhexidine (▲), hydrogen peroxide (*) and triclosan at MIC/8 (●). The optical density at which the samples were harvested is marked with an arrow.
